# Cost-Effective Open-Ended Coaxial Technique for Liquid Food Characterization by Using the Reflection Method for Industrial Applications

**DOI:** 10.3390/s22145277

**Published:** 2022-07-14

**Authors:** Eleonora Iaccheri, Massimiliano Varani, Luigi Ragni

**Affiliations:** 1 Department of Agricultural and Food Science, Alma Mater Studiorum, University of Bologna, 40127 Bologna, Italy; eleonora.iaccheri4@unibo.it (E.I.); luigi.ragni@unibo.it (L.R.); 2 Interdepartmental Center for Industrial Agri-Food Research, University of Bologna, 47521 Cesena, Italy

**Keywords:** liquid food, cheap device, S11, multivariate data analysis, reflection parameters

## Abstract

A cheap technique based on an open-ended coaxial probe together with a vector network analyzer was set up. The vector network analyzer *NanoVNA*, a very tiny handheld device, is the affordable component that gives the instrumental chain a cost-effective perspective. The open-ended coaxial probe is a cable with an SMA gold-plated termination. User-friendly programs can be used to calibrate the instrument, carry out the measurements, and save data on PC. Simple liquid solutions (sodium chloride, citric acids, and saccharose) and more complex liquid food (milk, egg products, and fruit juice) were investigated. In addition, the temperature on the electric measurement of milk was measured to evaluate a possible influence for refrigerated storage products. The reflection parameters, such as the real and imaginary parts of S11, were used to build univariate and multivariate models. The best results in terms of coefficient of determination and related error were 0.997 (RMSE 0.05%) for sodium chloride and 0.965 (RMSE 0.71 °Brix) for fruit juice considering the univariate model, and 0.997 (RMSE 0.04%) for sodium chloride and 0.981 (RMSE 4.44%) for yolk using multivariate analysis. The proposed solution is non-destructive, cheap, rapid, and very attractive for potential lab and industrial applications.

## 1. Introduction

Non-invasive detection techniques are of increasing interest to the food industry. They overcome several problems directly related to processing applications also being compatible online. Nevertheless, possible drawbacks can be represented by the high cost of the instrumentations and needing highly specialized employees [1]. An interesting opportunity for the development of non-destructive systems is given by the direct application of electromagnetism principles and statistical methodologies [2,3,4]. The response of a dielectric material interacting with an electromagnetic wave is related to the chemical-physical properties of the matter [5], applied frequency, and temperature.

Dielectric properties can be measured by several technologies available; however, high implementation costs limit the applicability of alternative instrumental chain. The dielectric characterization of liquid sample for quality parameters estimation can be achieved with time domain reflectometry (TDR), as example [5]. TDR can also be developed with components to set up a low cost instrumentation compared to the traditional solutions available in commerce [6]. However, it is a technique implemented with approximately at least a three thousand U.S. dollars [5,6], which is more expensive than that of the instrumental chain proposed in this work.

Other reflectometric techniques, such as infrared, neutron ray, and gamma ray sensors, have the advantage of contactless measurement, and they do not require calibration to the specific material, but they are very expensive and require special handling and caution for exploiting ionizing radiation [5,7].

Among all the techniques, the network analyzer coupled with the coaxial probe is a well-established technique concerning food analysis, and consider low cost, real-time, and non-destructive measurement with a quite high accuracy, as documented by the literature [1,2,8]. In addition, the open-ended coaxial probe provides more detailed information than that of TDR sensors [5,9]. The open-ended coaxial method is based on the scattering parameters measured as the sample response, particularly the reflection caused by impedance mismatch at the end of the line [9,10]. The dielectric properties of the sample are then derived from this impedance discontinuity, even though non-resonant methods are not the best for dielectric properties calculation [10]. Furthermore, consider that the liquid sample can be analyzed without any preparation requirement in a broadband capability [11].

Table 1 reports several devices developed with a coaxial line based on reflection principles considering food parameters estimation.

As Table 1 reports, the application of the coaxial probe technique is widely implemented for a wide range of purposes. Radio-frequency range, sometimes considering only the microwave portion, is the most used metric to measure food constituents, such as moisture content [12,13,14,15], alcohol [1,16], meat quality attributes [17,18], fruit maturity indexes and fruit juice parameters [19,20,21], acetic acid [22], fat in milk and vegetable oil [23,24], and hen eggs [25]. Different statistical analyses can be performed getting appreciable results in terms of coefficient of determination and error, as in Table 1, which shows pieces of evidence. The negative aspect of the technique is the price of the instrumental chain, which is expensive compared to other techniques, both regarding instrument (VNA, impedance meter) and commercialized probes and software. Li and co-authors [1] developed a cheap alternative to the probe, even if the network analyzer used is portable but still expensive or not identified. Concerning the above premises, the present research aims at developing and testing a very cost-effective instrumental chain for food parameter estimation based on a homemade open-ended coaxial probe and a portable, cheap vector network analyzer (*NanoVNA*). The tools used to date have a cost that exceeds thousands and if not tens of thousands of euros, while the proposed device is around one hundred euros, significantly reducing the cost and so the possibility of purchase by emerging countries. The reflection parameters, such as the real and imaginary parts of S11, were taken as predictors of several simple liquid solutions and complex food liquid matrices.

**Table 1:** Comparison of open-ended coaxial methods for non-destructive evaluation of food quality.

Sample	Range	Goodness of Estimation	Error	Device and Probe	Frequency	Cost of the Device	Author
Baijiu (chinese liquor)	40–56% alcohol by volume	R^2^ higher than 0.985	0.60%	Semi-rigid RG402 coaxial cable terminated with a female-type SMA (SubMiniature version A) connected to a FieldFox N9951A portable microwave analyzer (Keysight Technologies, Penang, Malaysia)	2–20 GHz	Probe: low cost Network analyzer: high cost	[1]
chickpea flour	7.9–20.9% moisture content	R^2^ 0.984–0.995	N.R.	An open-ended coaxial line probe connected to an impedance analyzer (HP4291B, Hewlett Packard Corp., Santa Clara, CA, USA)	10–1800 MHz	High cost (but N.R.)	[7]
Vidalia onions	8–91% moisture content	up to R^2^ 0.99	up to 1.3%	Agilent 85070E open-ended coaxial line probe connected to a 5230C PNA-L Network Analyzer	200 MHz–20 GHz	High cost (but N.R.)	[8]
Vegetable olis	Adulteration of olive oil with different percentages of sunflower oil 10–40%	PCA classification of the different samples	N.R.	Digital serial analyzer oscilloscope (Tetronix DSA 8200), equipped with a TDR module (Tektronix TDR 80E04)	20 Hz–1 MHz and 0–1.5 GHz	High cost (but N.R.)	[19]
Meat pork	Quality classes (Pale, Soft and Exudative (PSE), Dark, Firm and Dry (DFD) and Red, Firm and Non-exudative (RFN)	Classification with multifactorial ANOVA	95% confidence	Agilent 85070E Open-ended Coaxial Probe connected to an Agilent E8362B Vector Network Analyzer	500 MHz–20 GHz	High cost (but N.R.)	[12]
Meat pork after salting process	Samples were dipped in 25% NaCl solution for 0, 5, 10, 15, 25, 30, 40, 50, 60, and 90 min; 2, 2.5, 3, 4, 5, 8, and 12 h.	R^2^ 0.98	N.R.	Agilent 85070E Open-ended Coaxial Probe connected to an Agilent E8362B Vector Network Analyzer	500 MHz–20 GHz	High cost (but N.R.)	[14]
Rum Havana Club Anejo 3 Anos ~	Mixtures with methanol, ethanol, and deionized water	N.R.	3.3–6.3% range deviation values	PNA-X series Vector Network Analyzer (VNA) and the 85070-E model coaxial probe	500 MHz to 15 GHz	High cost (but N.R.)	[11]
Apple (Granny Smith) maturity	Maturity Index (MIdielectric) as a function of the Thiault Index (TI)	up to R^2^ 0.84	N.R.	Agilent 85070E Open-ended Coaxial Probe connected to an Agilent E8362B Vector Network Analyzer	500 MHz–20 GHz	High cost (but N.R.)	[26]
Milk	UHT whole, low fat and skim milk	N.R.	N.R.	Hewlett Packard 8510C Network Analyzer coupled to an 83651B Synthesized Sweeper, Agilent coaxial probe	1–20 GHz	High cost (but N.R.)	[18]
Butter	17–19% moisture content	up to R^2^ 0.97	N.R.	A vector network analyzer (Model: Agilent 8722ES, Agilent Technology, Palo Alto, Santa Clara, CA, USA) with an open-ended coaxial cable (#8120-6192, Hewlett Packard) connected to a probe (85070C, Agilent Technology, Palo Alto, Santa Clara, CA, USA)	500–3000 MHz	High cost (but N.R.)	[9]
Hen eggs	white albumin and yolk of eggs up to 15 days	up to R^2^ 0.999	N.R.	Hewlett–Packard 85070B open-ended coaxial probe connected to an Agilent 4291B Impedance Analyzer (Agilent technologies, Inc., Palo Alto, Santa Clara, CA, USA).	0.02–1.8 GHz	High cost (but N.R.)	[20]
Acetic acid	0–10%	R^2^ > 0.99	N.R.	A Hewlett–Packard 8510C network analyzer, coupled to an 83651B synthesized sweeper, and an 8517B S-parameter and Agilent 85070C coaxial probe	1–20 GHz	High cost (but N.R.)	[17]
Mexican sauces	permittivity as a function of moisture content, specific heat, viscosity, water activity, density, and electrical conductivity	N.R.	N.R.	Coaxial probe (Keysight Technologies, Santa Rosa, CA, USA) attached to a vector network analyzer (Keysight Technologies)	500 MHz–6 GHz	High cost (but N.R.)	[10]
pulque (Mexican traditional drink)	permittivity of different juice mixtures (natural, strawberry, and pineapple/coconut pulque)	N.R.	N.R.	A vector network analyzer, VNA (Keysight Technologies, N9918A FieldFox) and an 85070E open-ended coaxial probe kit	100 MHz–25 GHz	High cost (but N.R.)	[16]

R^2^ = coefficient of determination, N.R. = not reported.

## 2. Materials and Methods

### 2.1. Materials

Tests were carried out on simple water (deionized) solutions of sodium chloride, saccharose, and citric acid, and on more complex liquid food, such as cow milk, egg products, and fruit juices. In order to test the practical applicability in a real scenario, different brands of milk were selected. This can be traduced into different products characteristics as fat ranging between 0.1 and 3.8%, carbohydrates between 4.80 and 5.06%, protein between 3.2 and 3.4%, and minerals between 0.10 and 0.13%, as well as different geographical origin. Additionally, considering fruit juices, different brands with different taste and completely different formulations were evaluated, and are reported as follows: fat 0–0.5%, carbohydrates 2.9–15%, proteins 0–0.5%, and minerals 0–0.04%.

The simple solutions were analyzed to estimates the constituent by acquiring three replications of each concentration expressed in % of mass/volume (%, *m*/*v*). In total, 31 sodium chloride solutions (0.05 to 3.0% *m*/*v*), 40 solutions of saccharose (0.5–25.0% *m*/*v*), and 31 citric acid solutions (0.05 to 3.0% *m*/*v*) were examined. Cow milk was investigated for the estimation of fat percentages by using 37 kinds of milk with fat content, in the range of 0.1–3.8%. The yolk concentration in mixtures of albumen and yolk was estimated by considering 35 concentrations (0–100% of yolk). Finally, the sugar content in °Brix was estimated by using 9 fruit juices selected with a sugar content ranging from 3.5 to 15.1 °Brix.

### 2.2. Setup of the Device

**Figure 1:**
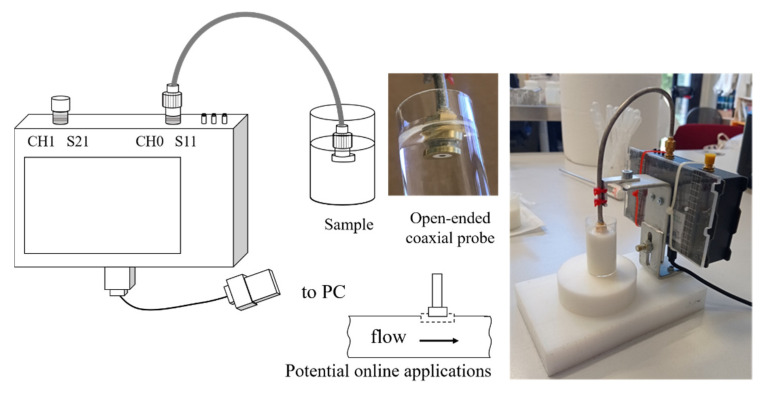
Layout and particulars of the instrumental chain.

A schematic layout together with images of the used instrumental chain is shown in Figure 1. 

The Vector Network Analyzer (VNA Nano V2, HCXQS in collaboration with China OwOComm) was selected as a very affordable and user-friendly component. The VNA is a two-port vector analyzer, with CH0 and CH1 ports for reflection and transmission detection, respectively. The device works in the radio frequency range of 50 kHz–3 GHz. In the present research, the whole frequency range was explored. The VNA was interfaced with the PC via USB and an own made coaxial probe. Coaxial cable offers the possibility to measure low frequency since it does not have the cut-off frequency typical of another kind of probe for reflection transmission characterization [26]. The probe was connected to the CH0 port. One port-port was used for the reflection signals evaluation. The device transmits and receives signals in a single port, allowing for the S11 reflection scattering parameter measurement. Therefore, S11 refers to the ratio between the amplitude of the reflected signal and the amplitude of the incident signal on port one, and it represents how much power is reflected from the antenna. S11 is represented by a complex number with a real and an imaginary part and it can be used to calculate magnitude and phase, more related to the permittivity able to explain the product dielectric behavior under the electromagnetic field. Complex permittivity can be calculated using the scattering parameters, as it happens for instrumentation based on VNA. Some works presented methods to calculate dielectric parameters by using S11 and known dielectric properties of several substances for calibration [2,8,27,28]. However, the determination of permittivity is beyond the scope of this work.

The probe was obtained by turning a female brass SMA connector into an electronic board. The probe is similar but improved to that suggested by Nuan-On et al. [28]. The active surfaces of the SMA probe were plated with a thin gold layer that was electrochemically deposed to avoid brass oxidation and loss in conductivity. A semirigid 50 Ohm cable was used to connect the probe to CH0.

The calibration has great importance for the device based on the reflection method as it can reduce the possible systematic errors characterizing one port measurements: directivity, source match, and reflection tracking [10]. Calibration was performed by using the commercial calibration kit SMA type (short, open, and 50 Ohm load, HCXQS in collaboration with OwOComm, China). Calibration and spectra acquisition was done with Nano-VNA-saver software (GNU, General Public License, version 0.3.8, Rune Broberg, https://github.com/NanoVNA-Saver/nanovna-saver/blob/main/LICENSE, accessed on 26 June 2022). The acquisitions were carried out in the whole frequency range, averaging three consecutive spectra, composed of 301 points (frequency step = 9.33 MHz). S11 reflection parameters were exported via Excel^®^ in the real and imaginary parts.

### 2.3. Data Analysis

For each of the simple solutions and complex liquid foods, the real and imaginary parts of the S11 scattering parameter were acquired. Each of the 301 spectral points was regressed to obtain a function that relates the S11 values to the reference parameter, accounting for the analysis of a couple of variables at a time.

Linear and quadratic regression between each averaged spectral point and the liquid food parameters were carried out to evaluate the goodness and robustness of the estimation using the coefficient of determination (R^2^), R^2^ adjusted, Root Mean Square Error (RMSE), and the significance (*p*-level) of the intercept and the angular coefficient.

Real and imaginary parts of S11 were also used, as average values of measurements replications as independent variables for multivariate investigation using Partial Least Squares regression analysis (PLS), by using Unscrambler software (Unscrambler software, version 9.7, CAMO, Oslo, Norway). The multivariate analysis accounts for all spectral variables and deals with the simultaneous relationship between them. PLS regression was conducted to set up predictive models for the constituent concentrations. Data were settled in samples of simple solutions and liquid foods (averaged values) × 301 (spectral points) matrix. The whole frequency range measured was explored, and loading weights were observed to understand which portion of the spectrum can give a higher contribution to explain the variability and for such reason can be more important for the model prediction power. Each group of samples was divided into calibration and validation sets. Then, 30% of the data set was randomly excluded in the training stage and used to validate the models. The calibration samples were used for computing the calibration models. Validation was then performed with unknown samples to characterize how the developed calibrated model would perform with new samples. Accordingly, test set validation was applied. The procedure was replicated three times, and the results in terms of coefficient of determination (R^2^), Root Mean Square Error (RMSE), and significative principal components (PCs) for calibration and validation were reported and discussed.

## 3. Results

**Figure 2:**
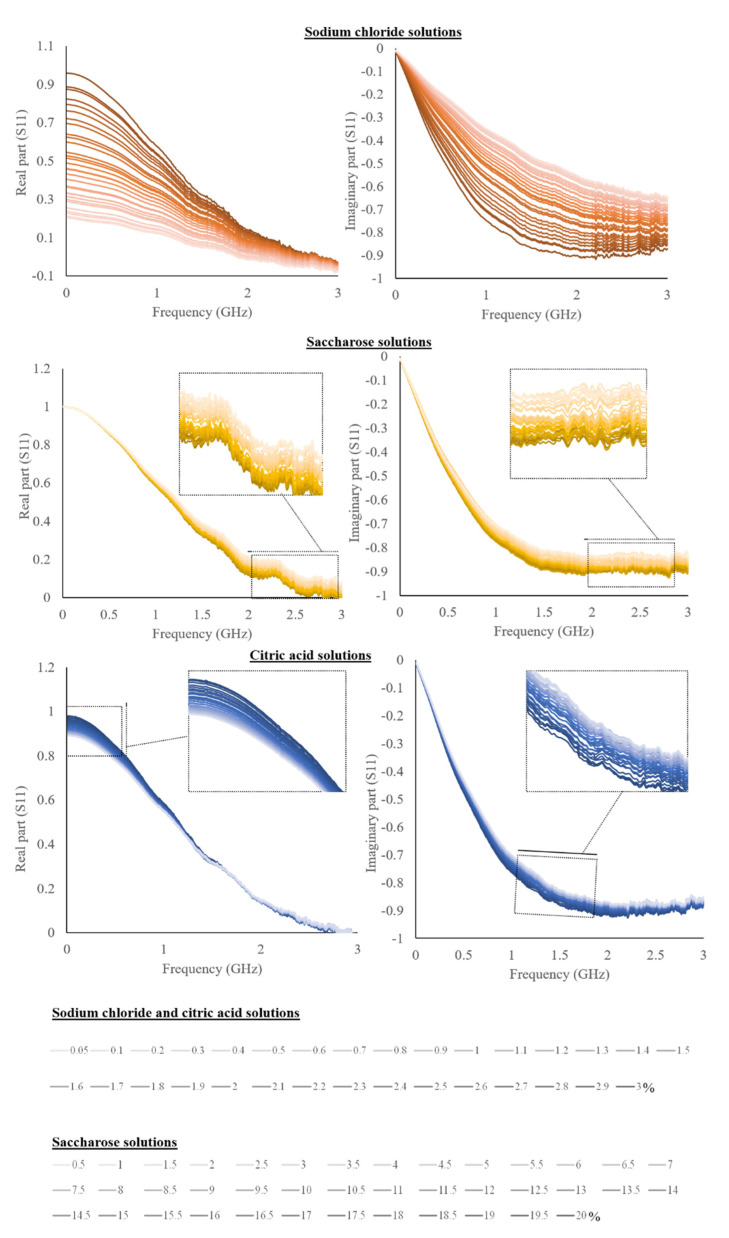
The real and imaginary part of S11 for sodium chloride, citric acid, and saccharose solutions (sodium chloride and citric acid have the same legend as the percentages are equal).

**Figure 3:**
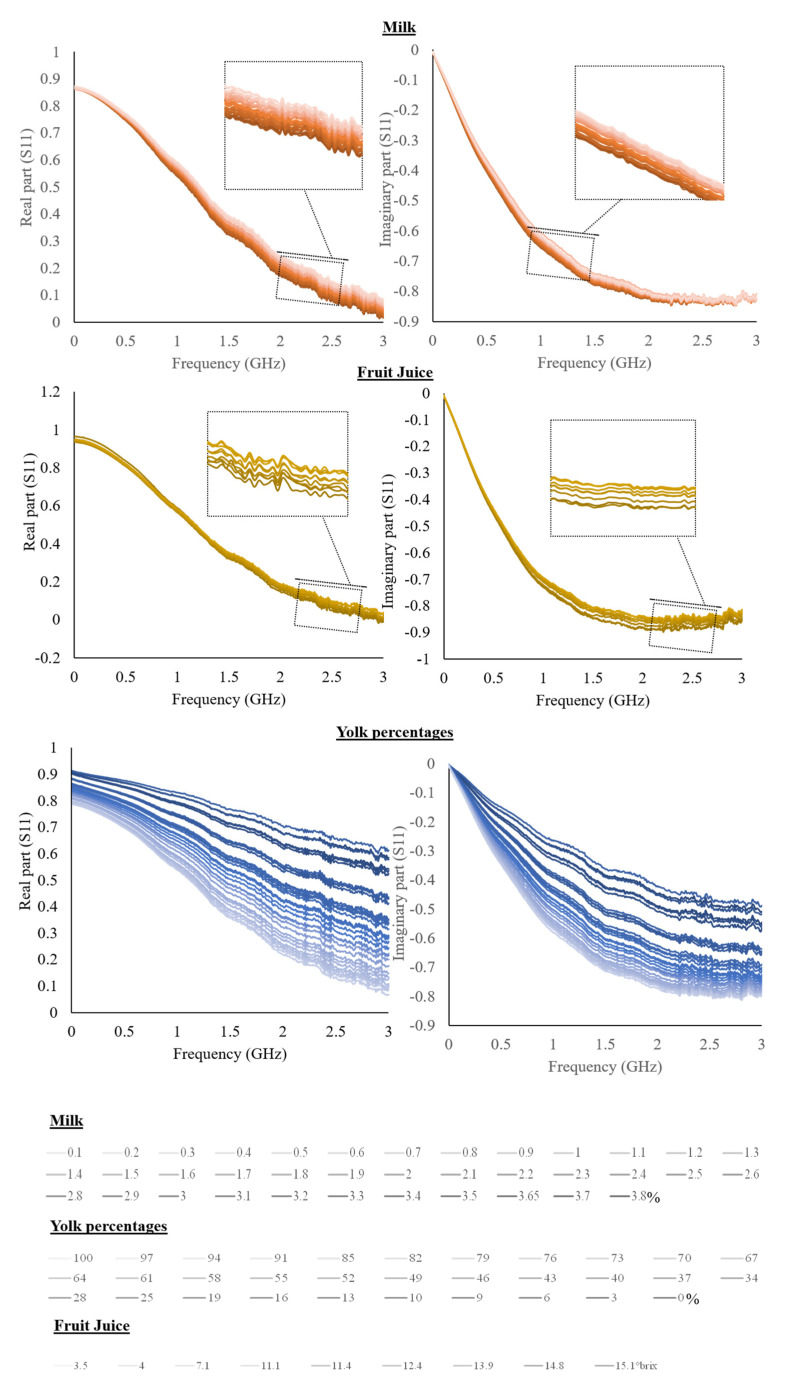
The real and imaginary part of S11 is a function of different milk fat content, yolk percentages, and °Brix of fruit juices.

Averaged spectra of the real and imaginary part of the S11 for the liquid solutions and liquid foods are shown in Figure 2 and Figure 3.

The figures show spectra variations as a function of the different concentrations of the investigated parameters.

As can be possible to observe from Figure 2, sodium chloride solutions showed a very huge spectral variability as a function of different concentrations than that of citric acid and saccharose. Despite this, the trend is visible for all substances. S11 real and imaginary parts are commented on with previous works by using real and imaginary parts of permittivity, also called the dielectric constant and loss factor, even if they are only indirectly related. Dielectric permittivity can be achievable but, as mentioned, it is not the focus of the work. The presented approach would like to be practical, cheap, and useful for laboratories or also industrial applications, for which the specific measure of dielectric permittivity is not required, and generally, it is customized for a defined set of materials [26]. Accordingly, dielectric permittivity references have been reported and considered as prior art, since previous works have a similar purpose, also considering the scarce data reporting the S11 reflection scattering parameter.

The real part of S11 of both sodium chloride and citric acid solutions decreases as a function of concentration and the opposite trend is revealed for the imaginary part. On the other hand, saccharose showed a decrease of both real and imaginary parts.

The effect of the solute in an aqueous solution is widely studied and it is caused by a decrease in permittivity compared to pure water [19]. This effect is accentuated as the concentration of the solute increases. Accordingly, the salt dielectric constant decreases as it bound the water [29].

Considering liquid food, the fat content in milk creates an increase in the intensity of the real part and a decrease in the imaginary part. The literature reports a slight decrease of dielectric constant as a function of fat by considering skim milk and milk with a high-fat content [30].

By increasing the percentage of the yolk of egg products, a decrease in both the real and the imaginary parts can be observed; the huge variation is dependent on the very large range investigated. Egg white and yolk were previously characterized by Kudra, obtaining values much closer to water when the egg white is considered than when the yolk is considered. Yolk samples had lower values of dielectric constant and loss factor [30]. Finally, as the °Brix of fruit juices increases, the imaginary part of the S11 increases, while the real part increases to about 1.2 GHz and subsequently decreases, showing a frequency dependence.

The literature reports the dielectric properties of juice modification as a function of °Brix. Particularly, the increase in concentration produced a decrease in the dielectric constant [20,31]. An important aspect to take into consideration for electrical measurements is the temperature [27]. In particular, milk was selected as it is a liquid food usually stored at refrigeration temperature. Accordingly, the influence of temperature on the electrical properties of milk was evaluated. Furthermore, the temperature has a recognized influence on electrical measurements, also with frequency dependence, and therefore affects the dielectric properties of food [12].

**Figure 4:**
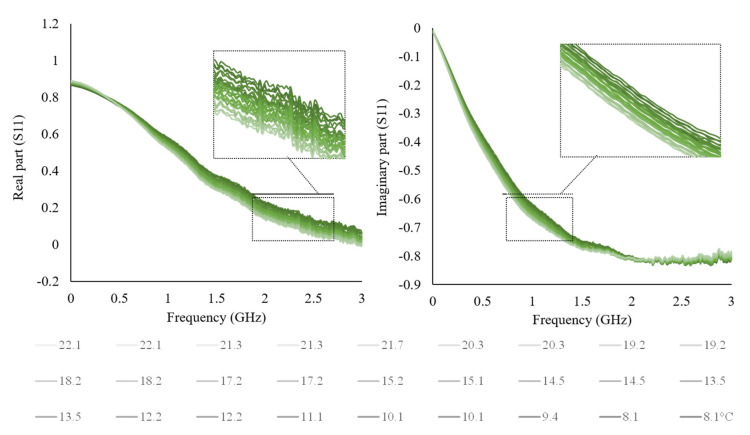
The real and imaginary part of milk as a function of temperature variation from 8.1 to 22.1 °C.

For this purpose, a single milk sample was considered to keep the composition constant and observe only the temperature influence. The sample was analyzed starting from the refrigeration temperature of 8.1 °C up to the ambient temperature of 22.1 °C, as shown in Figure 4.

The temperature measurements were made with a digital thermometer, at the same time as the electrical measurements. The real and imaginary part of S11 decrease as a function of temperature raise, and as expected, shows a frequency dependence (Figure 4). The temperature influence on waveforms could be overcome by applying a correction coefficient, carrying out temperature-dependent calibration models, or simply selecting the frequency range where the influence is negligible. This aspect could be helpful for sensor development, considering the milk production and storage temperature. Similar behavior is expected for the other used solutions and liquid foods since they contain a large amount of water.

Statistical parameters characterizing the analysis, as reported in the Section 2, are given in Table 2.

**Figure 5:**
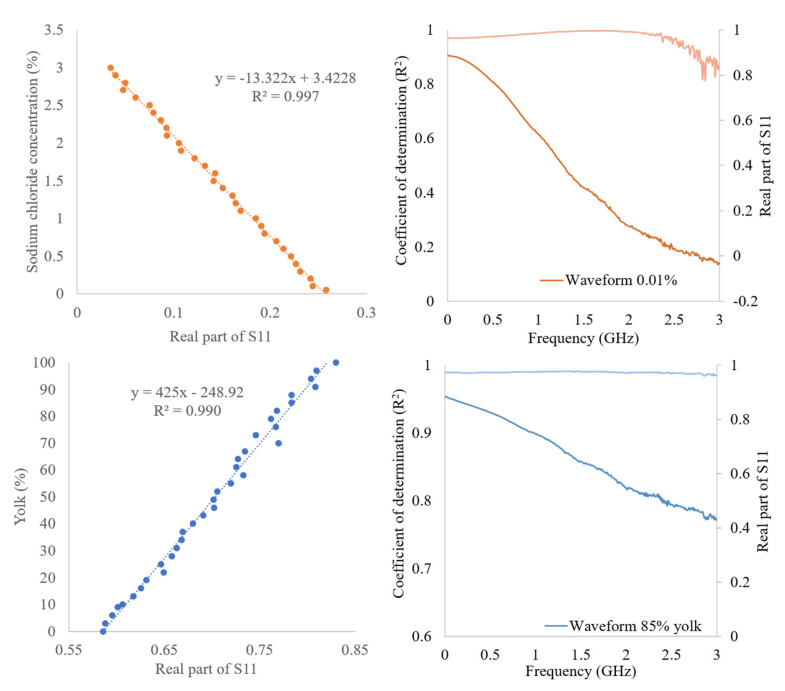
Best estimation fitting for sodium chloride and yolk % with related function (**left**) and a spectrum with a related coefficient of determination as example purpose (**right**).

Table 2 shows general linearity between the concentration of the analytes and the real and imaginary S11 values, except for citric acid, in which a quadratic trend is observed. The regression parameters of the equation having the best performance are reported in Table 2. The best point for each constituent is in the range in which there is the huge spectral variability. Even if the best point is reported, not only one point can contribute to the estimation, as demonstrated that the coefficient of determination is high for the most part of the spectrum (Figure 5).

The coefficients of determination and related RMSE indicate a good ability of the models to estimate the considered parameters. In particular, the greatest R^2^ was obtained for sodium chloride (0.997, RMSE = 0.05) considering the simple solutions and the yolk percentage in the egg product (0.988, RMSE = 3.29) both related to the real part. The empirical linear regressions were applied to calculate generic equations for device calibration, considering the selected simple solutions and liquid foods. Models obtained for different constituents showed low error values ranging from 1.6 and 6.8% calculated from the ratio of the RMSE on the total variation range, confirming the good accuracy of the proposed empirical equations.

For example, the best results in terms of the correlation coefficient are shown in Figure 5.

The figure also shows the fitting of the best point (at the determined frequency) between the real part of S11 and the constituent concentration. In addition, the R^2^ trend and a spectrum to underline the frequency portion with a better contribution to the estimation ability of the model are shown. As it can be seen, the coefficients of determination obtained remain high for the whole frequency range, confirming that a very good prediction is supported by a wide spectral region and not only a few points.

Data were also processed using multivariate techniques to consider the spectral information of the acquired waveforms. The real and imaginary parts of S11 were used as predictors for PLS model building to estimate the different parameters. The regression models were mainly carried out to predict concentration in future samples and for this reason, the selection of the test set validation was extremely important. Results are reported in Table 3 for calibration and validation as well. A good coefficient of determination (and low related errors) was generally obtained ranging from 0.933 to 0.998.

Results were comparable to bivariate regression analysis, even if it should be taken into consideration that PLS models were validated and therefore are more reliable. The loading weights were used to explore which frequency range can better contribute to the model prediction accuracy.

As confirmed also by simple regression, high loading weights were revealed for all the frequency range explored, so the whole was considered. Table 3 includes order information about the data set.

The proper number of PCs able to have the best estimation power is a crucial problem [32]. The selection of the PCs reported was done by observing Y-residuals validation variance (the plateau is 7 PCs) and selecting the maximum PCs number before the residual variance plot reaches the minimum.

However, some models resulted inappropriately as the variance was explained by a single main component. In this case, the bi-variate method proposed was found to be suitable and satisfactory to estimate the parameters of interest.

## 4. Conclusions

The assembled instrumental chain, based on a low-cost VNA and a handmade open-ended coaxial probe, was tested for the estimation of different food parameters in both simple solutions and more complex matrices. The univariate models, obtained from spectral points of the real or imaginary part of parameter S11, were characterized by coefficients of determination in the range between 0.965 (fruit juices) and 0.997 (sodium chloride). The relative RMSE errors were 0.75 °Brix for fruit juices (in a range from 3.5 to 15.1 °Brix) and 0.05% for the sodium chloride solutions (in a range from 0.05 to 3%). Multivariate PLS analysis was carried out giving a high coefficient of determination (from 0.933 to 0.998) for the test set validated models (30% external samples).

The proposed instrumentation is economical, simple, and easy to use and, thanks also to the small size of the probe, is suitable for small food quantities and online applications for process monitoring.

Future developments may include the elaboration of algorithms for the conversion of the reflection parameters S11 into dielectric constant and loss factor.

## Figures and Tables

**Table 2:** The coefficient of determination, adjusted coefficient of determination, RMSE, the frequency at which the function was obtained, the intercept’s significance (*p* level), and the angular coefficient for all the investigated solutions and food matrices.

Samples	Range of Variation	S11	Function	R^2^	R^2^ adj.	RMSE	Frequency (GHz)	Intercept Significance	Angular Coefficient Significance
Sodium chloride solutions	0.05–3%	R	y = −13.322x + 3.4228	0.997	0.997	0.05	1.70	<0.0000	<0.0000
		I	y = 12.86x + 11.092	0.991	0.991	0.08	3.00	<0.0000	<0.0000
Saccharose solutions	0.5–20%	R	y = 199.86x + 3.621	0.955	0.954	1.33	2.98	<0.0000	<0.0000
		I	y = 1.0137x +227.63	0.981	0.980	0.82	1.77	<0.0000	<0.0000
Citric acid solutions	0.05–3%	R	y = 267.93 × 2 − 530.83x + 262.99	0.994	0.994	0.07	0.90	<0.0000	(x2; x) <0.0000
		I	y = 600.33x2 + 1122.1x + 524.04	0.994	0.994	0.07	1.87	<0.0000	(x2; x) <0.0000
Milk (fat)	0.1–3.8%	R	y = 52.948x − 3.0812	0.987	0.986	0.13	2.61	<0.0000	<0.0000
		I	y = 122.34x + 46.584	0.984	0.983	0.14	4.60	<0.0000	<0.0000
Egg products (yolk)	0–100%	R	y = 274.47x − 106.16	0.988	0.988	3.29	1.39	<0.0000	<0.0000
		I	y = 679.4x + 207.72	0.982	0.982	3.97	0.42	<0.0000	<0.0000
Fruit juices (°Brix)	3.5–15.1°	R	y = 247.91x + 6.7011	0.969	0.965	0.71	3.00	<0.0000	<0.0000
		I	y = 305.04x + 267.56	0.965	0.960	0.75	2.99	<0.0000	<0.0000
Milk (temperature)	8.1–22.8 °C	R	y = −650.88x + 585.04	0.983	0.981	0.60	0.30	<0.0000	<0.0000
		I	y = −732.16x − 582.21	0.987	0.985	0.53	2.75	<0.0000	<0.0000

**Table 3:** Coefficient of determination in calibration and test set validation and a relevant number of principal components (PCs) of PLS models for all the investigated solutions and food matrices.

Sample		R^2^ Calibration	RMSE Calibration	R^2^ Validation	RMSE Validation	PCs
Sodium chloride	R	0.997	0.05	0.996	0.05	2
		0.997	0.05	0.996	0.05	2
		0.997	0.05	0.998	0.03	2
	I	0.996	0.05	0.997	0.05	2
		0.996	0.05	0.996	0.07	2
		0.996	0.05	0.997	0.05	2
Saccharose	R	0.978	0.76	0.989	0.70	3
		0.973	0.91	0.967	1.20	2
		0.983	0.76	0.982	1.09	3
	I	0.955	1.18	0.975	0.99	1
		0.957	1.22	0.975	0.82	1
		0.982	0.82	0.976	0.73	2
Citric acid	R	0.978	0.13	0.976	0.15	2
		0.987	0.10	0.948	0.23	2
		0.974	0.15	0.982	0.10	2
	I	0.968	0.16	0.966	0.15	2
		0.969	0.15	0.967	0.16	1
		0.969	0.16	0.968	0.16	3
Milk	R	0.981	0.14	0.990	0.12	1
		0.958	0.20	0.987	0.13	1
		0.986	0.14	0.952	0.17	1
	I	0.945	0.24	0.986	0.15	3
		0.979	0.15	0.954	0.27	4
		0.970	0.18	0.981	0.17	4
Yolk	R	0.958	5.90	0.984	4.12	3
		0.967	5.24	0.982	4.43	3
		0.965	5.52	0.978	4.76	3
	I	0.981	2.95	0.989	2.73	4
		0.988	3.21	0.972	3.32	3
		0.983	3.73	0.979	4.78	3
Fruit Juice	R	0.953	0.80	0.933	1.18	2
		0.996	0.20	0.974	0.86	4
		0.997	0.22	0.970	0.75	4
	I	0.969	0.40	0.960	1.35	3
		0.971	0.64	0.950	1.03	2
		0.891	1.21	0.991	0.51	1
